# Role of Mitochondria in Viral Infections

**DOI:** 10.3390/life11030232

**Published:** 2021-03-11

**Authors:** Srikanth Elesela, Nicholas W. Lukacs

**Affiliations:** 1 Department of Pathology, Michigan Medicine, Ann Arbor, MI 48109, USA; 2 Mary H. Weiser Food Allergy Center, Michigan Medicine, Ann Arbor, MI 48109, USA; nlukacs@med.umich.edu

**Keywords:** mitochondria, mitochondrial dynamics, viral infections, MAVS, RIG-I, MDA5, innate immune response, SARS CoV-2, RSV, influenza

## Abstract

Viral diseases account for an increasing proportion of deaths worldwide. Viruses maneuver host cell machinery in an attempt to subvert the intracellular environment favorable for their replication. The mitochondrial network is highly susceptible to physiological and environmental insults, including viral infections. Viruses affect mitochondrial functions and impact mitochondrial metabolism, and innate immune signaling. Resurgence of host-virus interactions in recent literature emphasizes the key role of mitochondria and host metabolism on viral life processes. Mitochondrial dysfunction leads to damage of mitochondria that generate toxic compounds, importantly mitochondrial DNA, inducing systemic toxicity, leading to damage of multiple organs in the body. Mitochondrial dynamics and mitophagy are essential for the maintenance of mitochondrial quality control and homeostasis. Therefore, metabolic antagonists may be essential to gain a better understanding of viral diseases and develop effective antiviral therapeutics. This review briefly discusses how viruses exploit mitochondrial dynamics for virus proliferation and induce associated diseases.

## 1. Introduction

Mitochondria are intracellular organelles that are considered as the powerhouse of the cell. They comprise an outer membrane, an inner membrane and a matrix. The highly complex metabolic process of conversion of carbohydrates and fatty acids to adenosine triphosphate (ATP) occurs in mitochondria. During cellular stress, mitochondria rapidly increase energy production [1]. Mitochondria have their own genomic DNA (mitochondrial DNA, mtDNA) and can replicate by using their own transcriptional machinery. [2,3]. Recent advances in the role of mitochondrial dysfunction in causing human diseases has led to an increasing number of studies targeting mitochondrial proteins, metabolic processes and subsequent signaling pathways for drug discovery. Mitochondria can sense inflammation, infection, and/or environmental insults through structural changes in mitochondrial membranes, and protein expression, resulting in dysfunction [4,5,6,7]. Mitochondrial dysfunction also affects metabolism, calcium regulation, airway contractility in lungs, gene and protein housekeeping, oxidative stress, cell proliferation and apoptosis. Dysfunctional mitochondria alter homeostatic cellular processes including aging and senescence [8], as well as airway diseases [9,10]. Thus, understanding how mitochondrial dynamics affect various disease conditions would open new avenues that enhance the development of novel therapeutics targeting dysfunctional mitochondria.

Viruses are obligate parasites that completely depend on host cell machinery for their replication and proliferation. They hijack host cell metabolism and cause substantial alterations in cellular and physiological functions [11]. The role of mitochondrial dynamics in viral infections is still emerging, but unequivocally depict mitochondria as a key for cellular metabolism and innate immunity, a promising avenue for further molecular investigations in viral pathogenesis. During viral infections, mitochondria are directly targeted by viral proteins or influenced by physiological alterations such as oxidative stress, hypoxia, endoplasmic reticulum stress (ER stress) and dysregulated calcium homeostasis [11,12]. A plausible metabolic link between mitochondria and influenza A, and with herpes viruses was shown in the 1950s by Ackerman and colleagues [13,14]. Recent studies showed how hepatitis B virus (HBV) [15,16] and hepatitis C virus (HCV) adopt the changes in mitochondrial dynamics for persistent infection [17,18]. Our laboratory has also shown that respiratory syncytial virus (RSV) infection affects mitochondrial function, leading to an altered immune response in lungs [19,20]. More investigations on the role of mitochondrial dynamics in viral pathogenesis will enhance our understanding of host–virus interactions, leading to the design and development of new antiviral strategies.

In this review, we discuss the importance of mitochondrial bioenergetics during specific viral infections and the impact on virus-induced mitochondrial dysfunction, leading to changes in innate immune responses. While there are common pathways that are affected during viral infections, such as inflammasome activation, we have separated individual viruses, as the observed changes in each virus may be distinct.

## 2. Mitochondrial Dynamics

Mitochondrial biogenesis is a complex process that involves coordination of both nuclear and mitochondrial genes to ensure precise function of proteins of the mitochondrial electron transport chain. Mitochondria are highly dynamic, but they cannot be generated de novo. Every mitochondrion consists of a porous outer membrane; the intermembranous space; an inner membrane where the electron transport chain (ETC) occurs; and a mitochondrial matrix, the main site for metabolic pathways such as the TCA cycle and fatty acid oxidation (FAO). Human mtDNA is a double-stranded, circular DNA molecule consisting of 16,569 base pairs [21]. The mitochondrial genome is comprised of 37 genes that encode 13 polypeptides (ETC essential genes), 2 rRNA genes (12S and 16S rRNA), and 22 tRNA genes required for mitochondrial protein synthesis [22]. The remaining mitochondrial proteins are encoded by nuclear genes and approximately 1500 nuclear encoded proteins are involved in regulating mitochondrial functions in humans [23,24].

**Figure 1:**
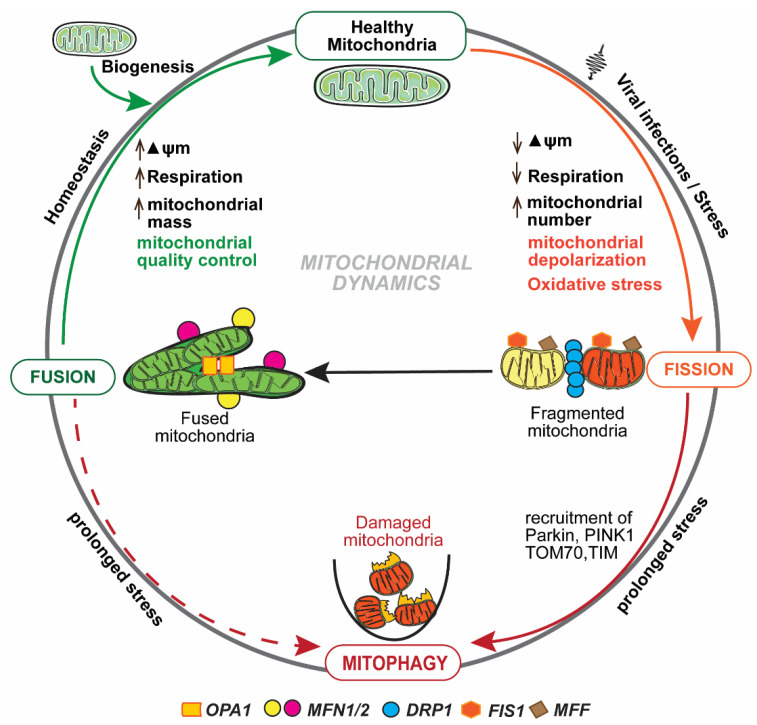
Mitochondrial dynamics: Mitochondrial fission and fusion are tightly regulated and continuous processes to maintain mitochondrial homeostasis. Fission is regulated by Drp1, Fis1, and Mff. Fusion is regulated by Opa1 and Mfn 1 and 2. Viral infections maneuver mitochondrial dynamics and alter mitochondrial membrane potential (ΔΨm), mtDNA function, and respiration rate. Interruption in any of these functions/pathways results in the accumulation of dysfunctional mitochondria that are eliminated by mitophagy.

Mitochondrial homeostasis is maintained predominantly by mitochondrial dynamics and mitophagy [25], as depicted in Figure 1. Mitochondria form a tubular network that continuously changes by fission and fusion [26], and both of these processes are regulated by large guanosine triphosphatases (GTPases) [27]. Fission and fusion are continuous processes, and dysfunctional or impaired mitochondria are eliminated by a tightly regulated process known as mitophagy [28]. Fission has been implicated in the correction of mutations in mtDNA copies [29], while fusion is involved in a swift exchange and equilibration of matrix metabolites and recycling of the partially impaired mitochondrion to a fully healthy mitochondrial network [25]. The irreversibly damaged mitochondria are selectively eliminated by mitophagy [28]. Mitochondrial fusion and mitophagy appear to have the same function yet are in fact distinctly complementary, and simultaneously play a critical role in mitochondrial homeostasis [25,26]. Mitochondrial membrane potential (ΔΨm) plays an important role in the process of identifying and segregating impaired mitochondria [30,31]. During a cellular cycle, the mitochondria follow a “kiss-and-run pattern” that promotes fusion for a very brief period (seconds), followed by a cellular change that shifts to fission [32]. Thus, the fission and fusion and mitophagy machinery work together to preclude circulation of impaired or dysfunctional mitochondria from the healthy pool of mitochondria. Quality and function of mitochondria are determined by the precise balance between incessant fission and fusion, and the subsequent processes that are induced manifest an enormous impact on the consequences of immune response during viral infections.

Viruses cause changes in mitochondrial function to promote viral translation and assembly. One theory is that virus–mitochondria interactions hamper mitochondria-associated antiviral signaling mechanisms [33]. In hepatitis viruses, mitochondrial antiviral signaling (MAVS) occurs through mitofusins that interact with MAVS to initiate effective antiviral immunity [34]. Blocking of mitofusins in cells resulted in the loss of mitochondrial membrane potential (ΔΨm), leading to defective antiviral immune responses, suggesting that mitochondrial integrity is essential for antiviral innate immunity. Similarly, increased ΔΨm induces apoptosis, while decreased ΔΨm prevents apoptosis, and viruses, such as human cytomegalovirus (HCMV), decrease ΔΨm to prevent cell death and promote their replication [35]. HCMV-encoded RNA (β2.7), localized in the mitochondria, interact with electron transport chain complex I and inhibit cells undergoing apoptosis [36], rapidly downregulating mitochondrial activity and enhancing viral replication. Virus-induced changes in mitochondrial integrity also result in an enhanced TCA cycle and further upregulation of lipid biosynthesis essential for viral envelopment, enlargement of the nucleus and of vesicular bodies of the infected cells [37,38]. During HCV infection, cells expressing HCV polyprotein have shown enhanced glycolytic function mediated by HIF-1α stabilization, with subsequently lower mitochondrial function, even in the presence of cellular oxygen, leading to increased cellular ATP content [39]. Increases in ATP levels have been reported in HCMV and herpes simplex-1 virus (HSV-1) infections [40]. Viral infections also induce ROS that control replication by altering mitochondrial function. In HCV infection, accumulation of defective mitochondria leads to oxidative stress and cell death [41]. Elevated ROS generation in the cells induces MAVS downstream, IRF3 and NFκB, to inhibit viral replication, linking a protective immune response with the virus infection. On the other hand, mitophagy decreases ROS production by removing dysfunctional mitochondria to control exacerbating immune responses [42]. The role of mitophagy in controlling viral replication is yet to be established. Nevertheless, it is interesting to note that mitophagy protects the cell from vulnerable cellular metabolic states. These viral effects are likely different depending on the virus itself and how it infects, replicates, and modifies innate immune cells. 

## 3. Mitochondrial Metabolism and Innate Immune Responses

Mitochondria originated from symbiotic bacteria but co-evolved with their host as most of the mitochondrial proteins are encoded by the nucleus. However, the mitochondrial genome encodes proteins critical for respiration. Mitochondria play a central role in cellular metabolism as key pathways such as TCA, FAO, oxidative phosphorylation (OXPHOS), calcium buffering, and heme biosynthesis occur in mitochondria [43]. It is well established that ATP is generated through oxidative phosphorylation [2]. Communication between the nucleus, mitochondria, and the cytosol is essential for the maintenance of proper mitochondrial function and cellular homeostasis [44]. Mitochondrial dysfunction has serious physiological consequences that led to pathogenesis of many neurodegenerative disorders, cancer, inflammation, metabolic syndrome, cardiac dysfunctions, and viral diseases [45,46]. Several studies have shown the role of mitochondria in the activation of the NLRP3 and NLRP6 inflammasomes, microbial- and host-derived metabolites, and metabolism that effects subsequent immune responses [47,48]. During infection, the activation of pattern recognition receptors (PRR) sends signals to mitochondria, which then shift the metabolic switch from oxidative phosphorylation to glycolysis in order to equip cells to effectively combat the pathogens [49], making mitochondria a primary target during microbial-triggered PRR activation. Several studies have shown that innate immune cells under various stimuli trigger unique metabolic signatures necessary for subsequent immune function [45,50].

Enzymes involved in metabolism are being extensively investigated due to similarities with immune regulators. Methylcrotonyl-CoA carboxylase 1 has been shown to be associated with TRAF6 and enhances MAVS signaling in order to induce antiviral type I IFN (interferon) secretion [51]. Type 1 IFNs were also shown to induce FAO and oxidative phosphorylation [52,53]. Metabolic intermediates of the TCA cycle such as succinate, fumarate and citrate are associated with various processes that are coupled with inflammatory pathways in both innate and adaptive immune cells. The preference of metabolic pathways for immune cells depends on several factors including cell type, differentiation state, activation conditions and the cellular microenvironment [45]. Macrophages stimulated with LPS and IFN prefer glycolysis, but when stimulated with IL-4, macrophages prefer OXPHOS and FAO to meet the energy demands of the cell [54]. Dendritic cells prefer glycolysis once they are infected and activated through PRR [44]. It is interesting to note that resting T lymphocytes and memory T lymphocytes rely on OXPHOS, but the proliferating T lymphocytes prefer glycolysis through the upregulation of glucose transporter glut-1 [55]. Neutrophils engage in glycolysis, including the release of neutrophil extracellular traps (NETs) with increased glut-1 ex-pression and glycolytic function [56]. Activated B lymphocytes undergo metabolic reprogramming as per bioenergetic and biosynthetic demands. Plasma cells are unique in that they take up more glucose and glutamine to potentiate both glycolysis and mitochondrial OXPHOS, essential for promoting cell survival [57,58]. The innate immune response has a critical role in both the detection and regulation of infectious insults. The recognition of the insult by PRR triggers specific innate immune cells and its respective receptor and ligand, also resulting in a swift reaction to additional immune cells of the disease. This early innate immune signaling is central to recognition of infecting viruses, stimulating recruitment of additional immune cells to the site, activating the specific adaptive immune response, and inducing the production of molecules necessary to combat infection for elimination of the infectious agent as well as repair of damaged tissues [59,60].

Mitochondrial structure and function can affect innate immune responses. The most direct effect of mitochondria on immune response is due to mitochondrial damage while it can also occur as a result of normal mitochondrial physiology and function. The innate immune system specifically recognizes pathogen-associated molecular patterns (PAMPs) and damage-associated molecular patterns (DAMPs) as alarmins in order to trigger the appropriate immune response [59]. The release of alarmins by mitochondria is due to cellular stress and loss of homeostasis. The DAMPs released by mitochondria include unmethylated CpG mtDNA [61], ROS [42], cardiolipin [62], and n-formyl peptides (n-fp) [63]. The exact mechanism by which these mitochondrial alarmins are released is still unknown. However, numerous studies have shown that it is mainly due to loss of mitochondrial membrane integrity. Importantly, these molecules are recognized by discrete receptors and trigger specific inflammatory pathways that restore normal cellular function.

## 4. Mitochondrial Antiviral Signaling (MAVS)

Viral genomes usually replicate in the host cell cytoplasm, where they are not recognized by TLRs such as TLR3, TLR7 or TLR8 due to TLR localization to endosomes [64]. However, RNA viruses can still be sensed in the cytosol through RIG-I-like receptors (RLRs) such as Retinoic acid-Inducible Gene-I (RIG-I), Melanoma Differentiation-Associated gene-5 (MDA5) and Laboratory of Genetics and Physiology 2 (LGP2). While RIG-I and MDA-5 are prototypical PRRs, LGP2 is a regulator of RIG-I and MDA5 signaling [65]. Both ssRNA and dsRNA are the known ligands of RIG-I and MDA5 [66]. RIG-I can also sense RNA polymers generated by the RNA polymerase III (pol III) from DNA templates, indirectly detecting dsDNA from intracellular pathogens [67]. RIG-I and MDA5 are cytosolic helicases with ATPase activity and consist of a regulatory C-terminal domain that binds to viral RNA, but the N-terminal domain comprises two tandem CARD domains (caspase activation and recruitment domains) [68]. The ATPase activity of these helicases is critical for translocation along dsRNA and in order to expose the CARDs that are masked by the C-terminal domain [69]. Once the specific 5′-triphosphate RNA structures are recognized, the E3 ubiquitin ligases TRIM25 and RIPLET enhance lysine 63-linked polyubiquitination of RIG-I, releasing CARDs from regulatory domain repression [70]. This conformational change leads to an essential interaction between the two CARD domains of RIG-I or MDA5 with the CARD domain of mitochondrial antiviral signaling protein (MAVS; also known as CARDIF, IPS-1 or VISA) [71,72]. MAVS, localized in the mitochondrial outer membrane, acts as a central signaling molecule in the RLR signaling pathway by linking upstream viral RNA recognition to downstream signal activation.

**Figure 2:**
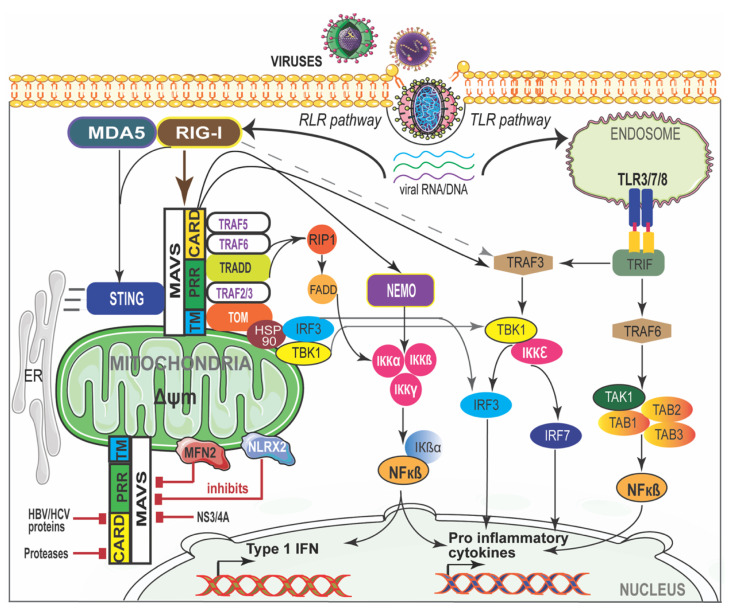
RIG-I/MDA-5 and MAVS interaction in viral disease. The cytosolic viral RNA/DNA is recognized by the RLR and/or TLR pathways. RIG-I-like receptors (RLRs) and MDA-5 activate MAVS through CARD and recruit signaling molecules to induce canonical nuclear factor-κB (NF-κB). NF-κB translocates into the nucleus and initiates pro-inflammatory cytokine gene expression. MAVS activates the stimulator of interferon genes (STING) and further mediates the activation of TANK-binding kinase 1 (TBK1) which phosphorylates interferon regulatory factor (IRF) signaling factors IRF-3 and IRF-7. IRF-3 then translocates into the nucleus and induces type I interferon (IFN) genes. NS3-4A, mitofusin 2 (MFN2), and NLR family member X1 (NLRX1) inhibit MAVS by preventing the formation of the MAVS–IKKi signaling complex. Hepatitis B virus (HBV) X protein promotes polyubiquitin conjugation of MAVS. ER—endoplasmic reticulum; MAM—mitochondria-associated membrane.

MAVS is required to localize to the mitochondria to exert its function, indicating that the mitochondrial environment is essential for signal transduction [71]. MAVS-deficient mice failed to induce type I IFN production and specific immune response against poly(I:C) suggests an essential role of MAVS in antiviral innate immunity [73,74]. RIG-I- and MDA5-mediated immune recognition and MAVS interaction are shown in Figure 2. Interaction between RIG-I or MDA5 with MAVS recruits a complex interactome to transduce the immune signaling. The MAVS-interacting proteins involved in antiviral response are TRAF3, TRAF5, IKKi/IKKε (IKKi), NEMO, DDX3, WDR5 IRF3, IRF7, and STING. The proteins that are involved in inflammatory responses are NLRC5, NLRX1, TRAF2, TRAF5, TRAF6, TAK1, and IKKα/β [75,76]. MAVS also interacts with mitochondrial proteins such as Mfn1, Mfn2, Tom70, and VDAC1; proteins involved in cell death (TRADD, FADD, RIP1) or autophagy (Atg5-Atg12); and with kinases (IKKi, PLK1, c-Abl, c-Src) or E3 ubiquitin ligases (PCBP2/AIP4, RNF5 and RNF125) that promote MAVS post-translational modifications [76,77]. Many of these proteins are indispensable and play a critical role in the canonical RLR pathway that is central to antiviral innate immune responses. Once activated, MAVS forms a signaling platform and recruits TNF receptor-associated factor (TRAF) 3 and TRAF6, inducing type I IFN [78] and inflammatory responses [79], respectively. TRAF3−/− cells have shown an impaired type I IFN response against viral infections [80]. TRAF3, along with NF-κB modulator protein NEMO [76], TRAF family member-associated NF-κB activator (TANK) [81] and NAK-associated protein 1 (NAP1) [82], regulates the activity of two noncanonical IKK-related kinases, TANK-binding kinase 1 (TBK1) and inducible IκB kinase (IKKi). The phosphorylation of interferon regulatory factors (IRFs), IRF3 and IRF7, by TBK1 and IKKi leads to the induction of type I IFN genes and a set of IFN-inducible genes that bind to IFN-stimulated response elements (ISREs) in the nucleus [83]. MAVS activates IRF3 through the ubiquitin-binding domains of NEMO, while NEMO itself activates TBK1 [84] through TRAF3 [85]. FAS-associated death domain-containing protein (FADD) was also found in a complex with MAVS that activates NF-κB downstream of MAVS through the FADD/caspase-8-dependent pathway [86]. TRADD, a tumor necrosis factor receptor (TNFRI) adaptor protein is recruited to MAVS and induces the activation of IRF3 and NF-κB by initiating a complex formation with TRAF3, TANK, FADD and RIP1 [87]. RIG-I-mediated activation of NF-κB requires MAVS and a complex of CARD9 and Bcl-10 adaptor proteins [88]. RIG-I also binds to the adaptor protein ASC and stimulates caspase-1-dependent inflammasome activation by a mechanism independent of MAVS, which suggests that RIG-I activates the inflammasome in response to certain RNA viruses [88,89,90].

## 5. Viral Infections and Mitochondrial Biogenesis

Viruses impede mitochondrial biogenesis, causing alterations in mitochondrial function in order to promote viral translation and assembly. One theory is that virus–mitochondria interactions hamper mitochondria-associated antiviral signaling mechanisms [33,91]. The regulation of mitochondrial dynamics in order to cause physiological perturbations in the cellular environment due to viral infections makes mitochondrial dynamics a primary target. Innate immune responses against viral infections led to type I Interferon (IFN-α/β) and other proinflammatory cytokines and chemokine responses. The specific molecules involved in mitochondrial biogenesis are peroxisome proliferator-activated receptor-γ coactivator (PGC)-1α, the main regulator of mitochondrial biogenesis [92,93]; PTEN-induced putative kinase 1 (PINK1) [94] that activates protein synthesis in damaged mitochondria; and the ligand-activated transcription factor aryl hydrocarbon receptor that functions to protect the cell from oxidative stress [95]. The silent information regulator-1 (SIRT1) activates the PGC1α-mediated transcription of nuclear and mitochondrial genes encoding for proteins during mitochondria proliferation, oxidative phosphorylation and energy production [96]. SIRT3, on the other hand, stimulates the proteins important for oxidative phosphorylation, the tricarboxylic acid cycle and fatty acid oxidation, and indirectly, PGC-1α and AMPK. SIRT1 deacetylates histone and numerous non-histone proteins during transcription, including PGC-1α [96]. Viruses have developed discrete strategies to regulate MAVS signaling by regulating mitochondria biogenesis, and thus regulating early innate immune responses.

### 5.1. SARS-CoV-2

A novel severe acute respiratory syndrome-related coronavirus (SARS-CoV-2) has recently emerged as a serious pathogen that causes high morbidity and substantial mortality. It is causing a global pandemic and worldwide social and economic disruption. Patients with severe SARS-CoV-2 infection develop dyspnea that can rapidly manifest as acute respiratory distress syndrome, leading to death [97,98,99,100]. SARS-CoV-2 is a single-stranded positive-sense RNA virus which encodes over 28 proteins, including 4 structural proteins (spike, membrane, envelope, and nucleocapsid), 16 non-structural proteins (NSP1–NSP16), and 8 auxiliary proteins (ORF3a, ORF3b, ORF6, ORF7a, ORF7b, ORF8, ORF9b and ORF14) [101,102]. The pathophysiology of SARS-CoV-2 infection shows exaggerated inflammatory responses, causing severe damage to the airways [103]. During SARS-CoV-2 infection in lungs, monocytes and macrophages are recruited to the site of infection and release cytokines and activate T and B cells. An impaired immune response during this process leads to chronic lung pathology. COVID-19 patients have shown dysregulated type I IFN response. However, the mechanisms by which SARS-CoV-2 evades host immunity have not been fully understood. SARS-CoV-2 M protein has been identified as a factor that interacts with MAVS to inhibit RLR-mediated induction of the host’s type I IFN response [104]. The M protein suppressed RIG-I-, MDA5- and MAVS-mediated signaling but did not show any effect on their downstream components TBK1 or p65. The authors have shown that the M protein directly interacts with MAVS and impairs viral RNA-induced MAVS through the downstream components TRAF3, TBK1, and IRF3 [104]. Screening of SARS-CoV-2 has identified several proteins including M, N, ORF3a, ORF6, and NSP (non-structural protein) family proteins as potential candidates that downregulate IFNβ responses [105]. Mitochondrial dysfunction-mediated reduced oxygen sensing, and mitochondrial oxidative stress-mediated platelet dysfunction and coagulation pathways have been reported in SARS-CoV-2 infection [106,107]. SARS-CoV-2 main protease Mpro (nsp5) impairs both the virus-induced type I IFN production and the induction of downstream antiviral interferon-stimulated genes (ISGs) [108]. Another protein, Orf9b, localizes to mitochondria, binds to TOM70, an adaptor protein of the mitochondrial outer membrane, and suppresses the antiviral type I IFN response [109,110]. However, the molecular consequences of Orf9b binding to TOM70 are not yet clear.

### 5.2. Respiratory Syncytial Virus

Human respiratory syncytial virus (RSV) of the Paramyxoviridae family is a single-stranded, negative-sense RNA virus that causes serious respiratory complications especially in infants and the older adults worldwide [111,112]. Quantitative proteomic analysis of RSV-infected cells has identified several nuclear-encoded mitochondrial proteins which include OMM complex subunits, respiratory complex I proteins, VDAC protein (voltage-dependent anion channel), and prohibitin (PHB) that play a critical role in the regulation of mitochondrial structure, function and biogenesis [113,114]. Hu et al. have shown for the first time that RSV infection hijacks host mitochondria, maneuvering for its replication and causing mitochondrial redistribution towards the perinuclear region of the microtubule organizing center [115]. This redistribution is a dynein-dependent mode of transport that causes perturbances in mitochondrial membrane polarization, leading to decreased mitochondrial membrane potential and significantly elevated levels of ROS [116]. Blocking dynein or the microtubule function resulted in a significant inhibition of RSV effect on mitochondrial function. In another study, deletion of a mitochondrial biogenesis factor, clustered mitochondria homolog (CLUH), resulted in enhanced mitochondrial ROS production during RSV infection [116]. The mitochondrial ROS scavenger MitoQ has been shown to remarkably reduce viral proliferation and restore mitochondrial function during RSV infection, suggesting that RSV-induced mitochondrial ROS contributes to sustained viral infection [115]. Similarly, our group has shown that SIRT1 is necessary to promote dendritic cell activation and autophagy during RSV infection, and the absence of SIRT1 led to exacerbated pathology [19]. In another study, we have also shown that mitochondrial function regulates RSV-induced innate immune response, leading to instruction of adaptive immune responses through SIRT1 [20]. The central role of acetyl coA carboxylase (ACC1) that activates acetyl CoA requires regulation by SIRT1 (via AMPK) in order to control the fatty acid synthesis pathway that leads to dysregulated innate cytokine responses. The inhibition of ACC1 has allowed the SIRT1-deficient dendritic cells to manifest a more appropriate innate and acquired immune response. The inhibition of ACC1 with a specific inhibitor led to correction of the altered metabolic state and resulted in the stabilization of the altered innate and acquired immune responses driven by RSV in DC and altered the pathologic responses in the lung [20]. However, the molecular mechanisms involving RIG-I/MDA5 and MAVS in RSV infection are yet to be explored.

### 5.3. Influenza Virus

Influenza virus is a respiratory pathogen that causes contagious respiratory illness known as influenza or flu, which accounts for millions of deaths worldwide. The three main types of influenza virus that cause disease in humans are A, B, and C, which are classified based on antigenic differences in matrix and nucleoproteins [116]. Once infected, the influenza virus is recognized by various PRRs such as TLRs, RIG-I, NLRP3, and cGAS pathways. The influenza virus replicates in the nucleus but how RIG-I signaling is activated during this is not very clear. However, the NS1 protein has been shown to suppress type 1 IFN responses by directly interrupting RIG-I signaling [117,118]. The nucleotide-binding oligomerization domain-containing protein 2 (NOD2) and receptor interacting protein kinase 2 (RIPK2) promotes ULK1 phosphorylation and induces mitophagy that protected mice from viral immunopathology in influenza A virus infection [119,120]. Defective mitophagy along with segregation of dysfunctional mitochondria and subsequent inflammasome activation was observed in RIPK2-depleted cells. Increased mitochondrial dynamics have been shown to downregulate IL-18 secretion and inflammasome activation [119,120]. Another protein, PB1-F2, disrupts mitochondrial membrane potential, binds to MAVS and downregulates innate immune responses, especially type 1 IFNs, and NLRP3 activation [121,122].

### 5.4. Hepatitis Viruses

Hepatitis C virus (HCV) is a positive-strand RNA virus of family Flaviviridae. During infection, HCV proteins localize to mitochondrial membranes, induce ER stress and cause depletion of ER calcium stores, leading to mitochondrial dysfunction [123,124]. The non-structural protein 5A (NS5A) of HCV inhibits electron transport chain enzyme complex I activity to promote mitochondrial calcium uptake, mitochondrial permeability transition, and ROS production [17,125]. NS3/4a protease, on the other hand, cleaves MAVS and facilitates immune evasion [126]. Mitochondrial damage during HCV infection inhibits FAO and enhances lipogenesis [127]. HCV induces translocation of Drp1 by phosphorylating it at S616 and promotes mitochondrial dynamics, subverts MAVS and increases IFN responses [17]. HCV infection induces the recruitment of Parkin and PINK1 and enhances the removal of accumulated impaired mitochondria in a Parkin-dependent manner [17]. Several studies indicate that HCV-induced regulation of mitochondrial dynamics favors viral persistence and illuminate how viruses exploit mitochondrial dynamics, leading to exacerbated pathology.

Hepatitis B virus (HBV) belongs to the family Hepadnaviridae and its genome consists of a partially double-stranded circular DNA that replicates via an RNA intermediate. HBx, a regulatory protein of HBV, is associated with VDAC, localizes to mitochondrial membranes and affects the membrane potential, inducing remarkably high levels of calcium and ROS, leading to mitochondrial dysfunction [128]. This HBx-regulated calcium signaling and ROS activate STAT3, NF-kB and NFAT [129]. Like HCV, HBV also induced Drp1 phosphorylation at S616 to promote mitochondrial dynamics and Parkin-mediated mitophagy [15]. Inhibition of Parkin during HBV infection increased the release of cytochrome C, activation of caspase-3, and cleaving of PARP (poly ADP-ribose polymerase), resulting in an enhanced apoptosis [130,131]. During infection, RIG-I in the cytosol detects HBV dsRNA in the cytosol [132], binds through its C-terminal RNA helicase domain and activates IKKi and TBK1 by CARD, which is at the N-terminal. MAVS then links RIG-I to IKKi and TBK1 activation. The role of MAVS/IPS-1 is essential for induction of IFN by cytosolic DNA [132,133].

### 5.5. Measles

Measles virus consists of a negative-sense RNA genome that causes highly contagious respiratory sickness including pneumonia, seizures, brain damage, and even death. The attenuated measles virus of the Edmonston strain (MV-Edm) activates p62-mediated mitophagy in non-small-cell lung cancer (NSCLC) cells by disrupting MAVS and resulting in the significant inhibition of type I IFN responses [134]. It utilizes apoptosis to sustain viral propagation and replication [135]. Defects in autophagy resulted in decreased viral titers and MV-Edm induced cell death in NSCLC cells. When p62 expression was silenced, it led to the restoration of mitochondrial mass in MV-Edm-infected cells and inhibition of mitophagy. Therefore, it appears that MV usurps mitophagy to mitigate the RIG-I/MAVS-mediated innate immune signaling pathways [134].

## 6. Concluding Remarks

Despite the exhilarating scientific advances in recent times that have identified many important metabolic pathways that might be targets in order to enhance immune responses, there continue to be new and exciting questions. Various aspects of mitochondrial dynamics, including mitophagy, have been of great interest, with recent studies showing that viruses circumvent host innate immune responses through altering mitochondrial functions. Viral infections induce metabolic re-programming, resulting in discrete bioenergetic phenotypes, strategically utilizing them for viral propagation and replication. Viruses exploit RIG-I-MDA5-MAVS antiviral signaling pathways and aim at disrupting mitochondrial membrane potential, mitochondrial-associated proteins and mitochondrial dynamics that essentially impede virus-induced type I IFN responses. Undeniably, there exists an intimate association between mitochondrial dynamics and viral infections. However, more comprehensive mechanistic studies and their significance to chronic pathology are necessary in understanding complex viral life cycle processes. A deeper understanding of tightly regulated mitochondrial functions such as bioenergetics, innate antiviral immunity, apoptosis, and inter-organelle cross-talk needs to be extensively investigated to analyze their effect on viral infections. The ultimate goal of identifying mechanisms, which may differ with individual viruses, may provide important information for targeted therapeutic interventions to redirect the immune response toward a less pathogenic response.
